# Supplementary material for: Exploring the factors influencing alarm fatigue in intensive care units nurses: A cross-sectional study based on latent profile analysis
Source: PLoS One. 2025 Jul 7;20(7):e0327644. doi: 10.1371/journal.pone.0327644 (PMC12233232; doi:10.1371/journal.pone.0327644)
Supplement: S1 Table — (DOCX) [file pone.0327644.s001.docx]

| **Variables** | **B** | **Std. Error** | **Beta** | **t** | ***P*** | **Tolerance** | **VIF** |
| --- | --- | --- | --- | --- | --- | --- | --- |
| (Constant) | 0.268 | 0.446 |  | 0.601 | 0.548 |  |  |
| Gender | 0.081 | 0.096 | 0.044 | 0.847 | 0.398 | 0.818 | 1.222 |
| Age | -0.104 | 0.063 | -0.129 | -1.654 | 0.099 | 0.358 | 2.790 |
| Education | 0.024 | 0.071 | 0.017 | 0.335 | 0.738 | 0.892 | 1.121 |
| Professiona | 0.091 | 0.048 | 0.139 | 1.898 | 0.059 | 0.405 | 2.467 |
| Position | 0.001 | 0.074 | 0.001 | 0.013 | 0.990 | 0.721 | 1.388 |
| Average monthly income | 0.037 | 0.051 | 0.040 | 0.726 | 0.468 | 0.726 | 1.377 |
| Number of children | 0.017 | 0.048 | 0.020 | 0.352 | 0.725 | 0.655 | 1.527 |
| Night shift frequency | -0.022 | 0.023 | -0.048 | -0.937 | 0.349 | 0.840 | 1.190 |
| Frequency of overtime work | -0.009 | 0.018 | -0.026 | -0.525 | 0.600 | 0.895 | 1.117 |
| Satisfaction | 0.051 | 0.052 | 0.054 | 0.990 | 0.323 | 0.731 | 1.367 |
| Health status and employee productivity score | 0.040 | 0.006 | 0.328 | 6.240 | ＜0.001 | 0.788 | 1.268 |
| Emotional labor score | -0.001 | 0.003 | -0.016 | -0.335 | 0.738 | 0.928 | 1.078 |

**S1 Table Multicollinearity Diagnostics for Independent Variables in the Regression Model**
