# Supplementary material for: Exploring the factors influencing alarm fatigue in intensive care units nurses: A cross-sectional study based on latent profile analysis
Source: PLoS One. 2025 Jul 7;20(7):e0327644. doi: 10.1371/journal.pone.0327644 (PMC12233232; doi:10.1371/journal.pone.0327644)
Supplement: Attachment — Submitted filename: Rviewers Comments (1).docx [file pone.0327644.s003.docx]

**INTRODUCTION**

**Strengths:**

Provides strong rationale: technological advancement → increase in alarms → alarm fatigue.

Cites well-known problems like false alarms, noise levels, and JCAHO data on adverse events.

Builds a solid case for investigating alarm fatigue in the Inner Mongolia region, highlighting the gap in regional studies.

**Suggestions for improvement**

The narrative occasionally drifts; some paragraphs are too long and unfocused.

“Given the differences in regional levels of economic development, healthcare resource allocation, and nursing work patterns, ICU nurses in Inner Mongolia may be unique in terms of the status of alarm fatigue.” could be rephrased as: “Variations in economic development, healthcare resources, and work patterns suggest that ICU nurses in Inner Mongolia face unique alarm fatigue challenges.”

Alarm fatigue in ICU nurses is closely related to personal traits, health status and employee productivity, and emotional labor.(could you provide reference for this) also you can Simplifying it could enhance readability. For example: *“Alarm fatigue among ICU nurses is influenced by personal traits, health status, employee productivity, and emotional labor.”* On the one hand, emotional labor is closely related to alarm fatigue in the work environment of ICU nurses, and excessive depletion of psychological resources affects the nurses' ability to process alarm information[14]. On the other hand, poor health status and employee productivity due to health problems can The phrase "emotional labor is closely related to alarm fatigue" is repeated. Consider combining ideas to avoid redundancy: *“Emotional labor, particularly in ICU environments, significantly contributes to alarm fatigue by depleting nurses’ psychological resources.”* it difficult for nurses to give full attention to their work and slow down the processing of alarm information, which may trigger or exacerbate alarm fatigue levels

The section could flow more smoothly by rephrasing the transition between the two main points (“On the one hand” and “On the other hand”).

“Spike alerts” should be “a spike in alerts.”

**METHODS**

Strengths:

- Sampling method, sample size estimation, inclusion/exclusion criteria are clearly explained.
- Instruments are validated and culturally adapted for the Chinese context.
- Detailed description of LPA and statistical indices used for model selection (AIC, BIC, entropy, LMR, BLRT).
- Ethical approval and informed consent are described.

**Areas of improvement:**

1.1Subjects of investigation should be Study participants, also in the first paragraph remove as survey respondents.

“The final inclusion of 597 clinical nurses in this study fully met the sample size requirement” could be rephrased as: *“The inclusion of 597 clinical nurses exceeded the minimum sample size requirement, ensuring a robust dataset.”*

Inclusion criteria of the survey respondents: (a) has certificate of nursing practice and registered; (b) working in ICU for≥1 year; exclusion criteria: (a) those who were in training or on leave during the survey period; (b) internships and rotating and seconded nurses; (c) nurses who suffered from significant life disasters in the last six months.

1.3 Certain sentences could be rephrased for smoother readability. For example:

*“Combined with relevant literature and survey needs designed by the researcher himself, it was used to collect demographic sociological information...”* could be revised to: *“Demographic information was gathered using a survey tool designed by the researcher, informed by relevant literature and study requirements.”*

1.3.4 “Chineseized” – non-standard term. Use “translated and validated in Chinese.”

1.4 Some sentences can be simplified for better readability. For example:

- *“Through the assistance of the nursing department of each hospital in Inner Mongolia Autonomous Region of China, the questionnaire star link or QR code was sent by the head nurse of each ICU unit to ICU nurses who met the inclusion criteria.”* could be revised to: *“The nursing departments of participating hospitals distributed the survey link or QR code through ICU head nurses to eligible participants.”*

**Data Exclusion Criteria:**

- While invalid responses are excluded, providing a rationale for choosing <2 minutes as the threshold for invalidity would enhance clarity.

1.5 Some sentences are overly technical and could be streamlined for readability. For example:

- *“Given the nature of the data, with some variables being ordinal and others not meeting the assumption of normality, non-parametric rank-sum tests (Kruskal-Wallis H test) were utilized to compare differences in general demographic characteristics across the identified latent profile categories.”* could be rephrased as: *“The Kruskal-Wallis H test was used to compare differences in demographic characteristics across profile categories, addressing ordinal variables and non-normal distributions.”*

**RESULTS**

Strengths:

- Participant characteristics are well-detailed and geographic spread is reported.
- LPA model selection process is sound and well-supported by fit indices.
- Clear distinction among three alarm fatigue profiles.

**DISCUSSION**

Strengths:

- Interprets the profiles meaningfully and links findings to workload, emotional labor, and presenteeism.
- Connects with prior studies and literature, including cross-national findings.
- Offers practical implications for nurse managers (training, scheduling, support).

Areas of improvement:

Some parts are repetitive or written in an overly prescriptive tone (“administrators should…”).

Emotional labor and cognitive load sections are strong but could be more concise.

Limit redundancy in repeating what was already stated in results.

3.1	Some sentences are long and could benefit from simplification. For example:

- *“On the other hand, invalid alarms and false alarms are frequent in alarm messages, which interferes with nurses' judgment and work efficiency and reduces their sensitivity to alarm signals [26].”* could be revised to: *“Frequent false and invalid alarms disrupt nurses' judgment, decrease work efficiency, and reduce their sensitivity to alarm signals [26].*
- The transition between general alarm fatigue findings and specific profile categories could be smoother. For example, you could add a linking sentence like: *“To better understand these findings, the study categorized ICU nurses into three distinct profiles based on their alarm fatigue levels.”*

Rephrasing the transition between the two main points (“On the one hand” and “On the other hand”).

3.2.1 Certain sentences could be streamlined for clarity and readability. For instance:

- *“On the one hand, a large part of the group of childless nurses are young nurses with short working time and relatively little clinical experience.”* could be rephrased as: *“Many childless nurses are young, with limited work experience and clinical exposure.”*

3.2.2 Certain sentences could be refined for better readability. For example:

- *“And this study further found that nurses with a night shift frequency of 1-4 times per month had more severe alarm fatigue than nurses with a night shift frequency of >4 times per month.”* could be revised to: *“This study revealed that nurses working 1-4 night shifts per month experienced greater alarm fatigue compared to those working more than 4 night shifts monthly.”*

3.2.3 Some phrases could be simplified for clarity. For instance:

- *“Health status and employee productivity is a state in which nurses are formally present at work, but essentially fail to fully utilize their work capacity due to physical and mental health problems.”* could be revised to: *“Health status and employee productivity reflects nurses who are physically present at work but cannot fully perform due to health challenges.”*

**Overall Comment on the Manuscript**

This manuscript provides a comprehensive exploration of alarm fatigue among ICU nurses, utilizing latent profile analysis to categorize fatigue levels and investigate influencing factors. The study makes valuable contributions to understanding a critical issue in ICU management and offers actionable recommendations for reducing alarm fatigue and improving nurse well-being. However, there are areas where refinement and additional context could enhance the manuscript's clarity, impact, and accessibility.

**Clarity and Readability:**

- Some sections, particularly the discussion and results, contain dense and lengthy sentences. Streamlining the language and simplifying technical jargon would make the manuscript more accessible to a broader audience.
- **Formatting and Language Consistency:** Standardizing terminology (e.g., “childless nurses” versus “nurses without children”) and improving transitions between sections would enhance the manuscript’s flow and coherence.
