# Supplementary material for: Exploring the factors influencing alarm fatigue in intensive care units nurses: A cross-sectional study based on latent profile analysis
Source: PLoS One. 2025 Jul 7;20(7):e0327644. doi: 10.1371/journal.pone.0327644 (PMC12233232; doi:10.1371/journal.pone.0327644)
Supplement: Attachment — Submitted filename: Response to Reviewers.docx [file pone.0327644.s005.docx]

**Response to Reviewers**

**Manuscript ID:PONE-D-25-11224R1**

**Title:Exploring the factors influencing alarm fatigue in intensive care units nurses: a cross-sectional study based on latent profile analysis**

Dear Dr. Othman A. Alfuqaha and Reviewers,

We extend our heartfelt gratitude for the meticulous review and insightful feedback provided on our manuscript titled, "Exploring the factors influencing alarm fatigue in intensive care units nurses: A cross-sectional study based on latent profile analysis." The professionalism and efficiency demonstrated throughout this process have reinforced our pride in submitting to PLOS ONE.

In response to the reviewers' comments, we have conducted a thorough review and implemented all required revisions with the utmost diligence. All modifications have been meticulously addressed to align with the journal's standards and the reviewers' recommendations. The revised manuscript has been updated accordingly, with all changes carefully incorporated.

We are particularly appreciative of the constructive suggestions provided, which have significantly enhanced the clarity, rigor, and overall quality of our work.Should any further clarifications or additional materials be required, please do not hesitate to contact us. Once again, we express our sincere appreciation for your time, expertise, and the invaluable contributions to improving our manuscript.

Thank you for considering our work for publication in PLOS ONE. We look forward to your continued feedback.

Sincerely,

Yanling Chen (Corresponding Author)

On behalf of all co-authors

**❤ Response to Editor**

Dear Dr. Othman A. Alfuqaha ,

We extend our heartfelt gratitude for the meticulous review and insightful feedback provided on our manuscript. The professionalism and efficiency demonstrated throughout this process have been truly commendable, and we are deeply appreciative of the constructive suggestions that have significantly enhanced the quality of our work.

In response to the comments, we have conducted a thorough review and implemented all required revisions with the utmost diligence. **Regarding the references, we have re-examined all references, ensuring they have been retrieved from credible sources such as PubMed and are in good standing without any anomalies.** The formatting of the references has also been carefully checked to conform to the guidelines of PLOS ONE.

We would also like to respectfully request a revision to the authorship order of our manuscript. Based on the substantial contributions made by Ms. Wenxia Zhang during the major revision phase, we propose to list her as a co-first author alongside the original first author. The updated authorship order would be: Liya Li*, Wenxia Zhang*, Yanling Chen. (* denotes equal contribution). Previously Ms. Wenxia Zhang was the second author of the article. And I have made change in the revised manuscript. Hopefully, we can get your approval and consent.

The rationale for this revision is as follows:

• **Contribution to Revisions:** Ms. Zhang made pivotal contributions during the revision process, including calculating reliability, revising the limitations of the article, addressing multicollinearity, proofreading the original data, and drafting the response letter to the reviewers.

• **Critical Intellectual Input:** Her revisions significantly enhanced the clarity and scientific rigor of the manuscript, particularly in the Discussion section.

• **Language Polishing:** She also undertook comprehensive language editing, greatly improving the readability of the paper.

We have carefully reviewed the journal's authorship guidelines and confirm that we are in compliance with all requirements. To facilitate this change, we have completed the **PLOS_Authorship_Change_Form** and have attached a scanned copy of the **Written approval from all authors**, including their signatures consenting to this revision. I have uploaded two PDFs into the system.

We understand that authorship order changes may raise concerns, and we apologize for not addressing this initially due to our unfamiliarity with the journal's policies regarding authorship changes. However, after a careful review of the guidelines, we believe it is appropriate to formally request this revision.We have carefully reviewed the journal's authorship guidelines ([ Editorial and publishing policies-Authorship]) and confirmed that ( <https://journals.plos.org/plosone/s/authorship> ): *All authors should be listed in the Editorial Manager submission form before peer review. If a submission’s authorship changes during peer review or revisions, the author fields in the Editorial Manager submission form should be updated at the next available opportunity (e.g. when submitting the revision), and authors must fulfill the requirements described here.*

**Should the journal not accommodate this revision, we respectfully accept the decision and will proceed with the original authorship order.**

Once again, we express our sincere appreciation for your time, expertise, and the invaluable contributions to improving our manuscript. Thank you for considering our work for publication in PLOS ONE.

Warm regards,

Yanling Chen (Corresponding Author)

On behalf of all co-authors

❤ **Response to Reviewer #1:**

On behalf of all the authors, I would like to extend our heartfelt gratitude for your meticulous review and insightful feedback on our manuscript titled, "Exploring the factors influencing alarm fatigue in intensive care units nurses: A cross-sectional study based on latent profile analysis." We deeply appreciate your professionalism and efficiency throughout the review process.

After the last major revision, I learned a lot as well as recognized the shortcomings of the manuscript. We understand that a high-quality manuscript requires careful scrutiny and revision. We are truly grateful for your invaluable comments, which have not only helped us improve the manuscript but also broadened our research perspectives.

We have carefully addressed all your suggestions and made further improvements to ensure the manuscript meets the high standards of PLOS ONE. Should you have any further questions or suggestions regarding this revision, please do not hesitate to contact us. We would like to express our sincere gratitude once again for your hard work during the review process. We eagerly await your feedback and hope that our manuscript will be deemed suitable for publication in PLOS ONE, contributing to the research on alarm fatigue among ICU nurses.

★ **Please review the PLoS ONE guidelines for font (12-point Times New Roman) and subheadings to ensure consistency throughout the manuscript.**

**➦Response**

Thank you for your meticulous review and for bringing the formatting inconsistency to our attention.

Upon receiving your comment, we conducted a thorough review of the entire manuscript, specifically focusing on the font size and typeface. We identified that the abstract and body text were indeed formatted in 10.5-point Times New Roman, which is not consistent with the journal's requirement of 12-point Times New Roman.

**We have carefully corrected this discrepancy and ensured that the abstract and body text throughout the manuscript are now uniformly formatted in 12-point Times New Roman font.** Furthermore, we have double-checked the formatting of all headings and subheadings (Level 1, Level 2, and Level 3) to confirm they adhere to the journal's specified styles, and we can confirm that no further corrections are needed in this regard.

A revised version of the manuscript, incorporating these formatting adjustments. We have also highlighted these changes in the "Track Changes" version for your easy reference.

We are grateful for your vigilance in ensuring the manuscript meets the high standards of PLOS ONE. Your expertise and attention to detail are greatly appreciated. Please let us know if you require any further revisions or have additional comments.

★ **Methods Section**

**- As your study adheres to the STROBE guidelines, please incorporate the recommended structure, including a "Study Design" subsection.**

**➦Response**

Thank you for your meticulous review and for bringing the importance of adhering to the STROBE guidelines to our attention. We sincerely appreciate your expertise and guidance in ensuring the quality and transparency of our research reporting. We have carefully considered your comment regarding the incorporation of a "Study Design" subsection in our manuscript, in line with the STROBE guidelines.

In addition, we read and browsed plos one papers related to the nursing profession published within the last year, for which the methods section was considered. Based on your valuable suggestions, we have modified the secondary heading of the “Materials and methods” section of the manuscript.

We believe this revised structure enhances the flow and clarity of the Methods section, while ensuring compliance with the STROBE guidelines. By incorporating the "Study design and participants" subsection, we have aimed to provide a more integrated and transparent account of our study's methodology. The research design of this study is described in section 1.1.

We are committed to ensuring the highest standards of reporting in our research and are open to making further revisions if necessary. Thank you again for your time and effort in reviewing our manuscript. We deeply appreciate your valuable contributions to improving the quality of our work.

**➦Comparison before and after modification**

**Before modification: 1.1 Study participants**

**Changed: *1.1 Study design and participants***

**Before modification: 1.3 Survey instruments**

**Changed:** ***1.3 Measurement instruments***

★ **Discussion Section**

**- Consider removing subsections to improve the flow and coherence of the discussion.**

**➦Response**

Thank you for your meticulous review and thoughtful suggestion regarding the Discussion section of our manuscript. We sincerely appreciate your expertise and guidance in improving the flow and coherence of our manuscript.

We agree with your valuable comment that removing subsections from the Discussion section could enhance its readability and logical flow. After careful consideration, we have deleted the four tertiary headings (3.2.1, 3.2.2, 3.2.3 and 3.2.4) from the discussion section. And the body and order of the Discussion section remain unchanged. We believe this revision has indeed improved the overall coherence of the Discussion, allowing for a smoother and more integrated presentation of our findings and interpretations.

The revised Discussion section now presents a more continuous narrative, focusing on the key findings regarding the factors influencing alarm fatigue among ICU nurses and their implications for nursing management.

We are grateful for your careful review and constructive feedback, which have significantly contributed to the refinement of our manuscript. We believe these revisions have strengthened the overall quality and impact of our work.

**➦Delete content**

3.2.1 Number of children

3.2.2 Frequency of night shift

3.2.3 Health status and employee productivity

3.2.4 Emotional labor

★ **References**

**- Verify that the references are formatted correctly in 12-point Times New Roman font.**

**➦Response**

Thank you for your meticulous review and for highlighting the importance of proper reference formatting. We sincerely appreciate your careful attention to detail and your commitment to maintaining the high standards of PLOS ONE.

We have carefully reviewed your comment regarding the font size of the references. We would like to confirm that we have diligently addressed this concern by changing the font size of all references from 10.5-point to 12-point Times New Roman, as per the journal's guidelines.

Furthermore, we have conducted a thorough re-examination of the entire reference list to ensure that all formatting requirements are met.

We are grateful for your vigilance in ensuring the manuscript adheres to the journal's formatting standards. Your expertise and attention to detail are greatly appreciated. Please let us know if you require any further revisions or have additional comments.

★ **Tables and Figures**

**- Add p-values where necessary in the tables.**

**- Enhance the clarity of the figure to improve readability.**

**➦Response**

Thank you for your meticulous review and for your valuable suggestions regarding the tables and figures in our manuscript. We have carefully considered your comments regarding the addition of *P*-values and the enhancement of figure clarity.

In response to your suggestion regarding the tables, we have conducted a thorough review of all tables, with particular attention to Table 3. We identified that the presentation of *P*-values in Table 3 was not entirely consistent with the recommended formatting. To address this, we have revised the table to clearly display "*P*-value" instead of just "*P*," ensuring consistency and adherence to standard statistical reporting practices.

Regarding the figure, we have taken your comments regarding readability very seriously. To ensure our figure meet the high standards of PLOS ONE, we utilized the journal's recommended online tool (PACE) to rigorously review and enhance the formatting of figure. This process included optimizing image resolution and ensuring compliance with the specified dimensions. We can confirm that the pixel dimensions of the figures are now 1002*653, fully meeting the requirements outlined in the journal's guidelines.

Thank you again for your time and effort in reviewing our manuscript! We deeply appreciate your valuable contributions to improving the quality and clarity of our research!

**➦Comparison before and after modification**

**Before modification:**

Table 3. Results of multivariate logistic regression analysis (N = 582).

| **Variable** | **Low fatigue-robust tolerance group(ref. high fatigue-negative coping group)** | | | | | | **Moderate fatigue group(ref. high fatigue-negative coping group)** | | | | | |
| --- | --- | --- | --- | --- | --- | --- | --- | --- | --- | --- | --- | --- |
|  | **B** | **SE** | **OR** | **95%CI** | | ***P*** | **B** | **SE** | **OR** | **95%CI** | | ***P*** |
|  |  |  |  | **L** | **U** |  |  |  |  | **L** | **U** |  |

**Changed:**

| **Variable** | **Low fatigue-robust tolerance group(ref. high fatigue-negative coping group)** | | | | | | **Moderate fatigue group(ref. high fatigue-negative coping group)** | | | | | |
| --- | --- | --- | --- | --- | --- | --- | --- | --- | --- | --- | --- | --- |
|  | **B** | **SE** | **OR** | **95%CI** | | ***P-*value** | **B** | **SE** | **OR** | **95%CI** | | ***P*-value** |
|  |  |  |  | **L** | **U** |  |  |  |  | **L** | **U** |  |
